# Distribution of *papG* alleles among uropathogenic *Escherichia coli* from reproductive age women

**DOI:** 10.1186/s12929-022-00848-5

**Published:** 2022-09-07

**Authors:** Timothy Kudinha, Fanrong Kong

**Affiliations:** 1 NSW Health Pathology, Regional and Rural, Orange Base Hospital, Orange, NSW 2800 Australia; 2 School of Biomedical Sciences, Charles Sturt University, Orange Campus, 346 Leeds Parade, Orange, NSW 2800 Australia; 3 Centre for Infectious Diseases and Microbiology Laboratory Services, NSW Health Pathology, Westmead Hospital, Westmead, NSW 2145 Australia

**Keywords:** Uropathogenic *Escherichia coli*, Virulence genes, *papG* alleles

## Abstract

**Background:**

Extraintestinal *Escherichia coli* (*E. coli*) causing urinary tract infections (UTIs), and often referred to as uropathogenic *E. coli* (UPEC), are a major contributor to the morbidity of UTIs and associated healthcare costs. UPEC possess several virulence factors (VFs) for infecting and injuring the host. We studied the *papG* allele distribution, and its association with other VF genes and phylogenetic groups, amongst 836 UPEC and fecal isolates from reproductive age women.

**Results:**

The *papGII* gene was highly prevalent amongst pyelonephritis isolates (68%), whilst the majority, *albeit* smaller proportion, of cystitis isolates (31%) harboured the *papGIII* gene. Among the pyelonephritis and cystitis isolates, *papG* positive isolates on average had higher VF gene scores, and were more likely to belong to phylogenetic group B2, than their negative counterparts. This was mostly due to the contribution of *papGII* isolates, which on average contained more VF genes than their *papGIII* counterparts, irrespective of the uro-clinical syndrome. However, the *papGII* isolates from the pyelonephritis cohort had higher VF gene scores than the cystitis ones, suggesting presence of possible *papGII* clones with differing inferred virulence potential. Furthermore, *papGII* isolates were more likely to possess an intact *pap* gene operon than their *papGIII* counterparts. Also of note was the high proportion of isolates with the *papGI* allele which was not associated with other *pap* operon genes; and this finding has not been described before.

**Conclusions:**

The association of the *papGII* gene with several VF genes compared to the *papGIII* gene, appears to explain the abundance of these genes in pyelonephritis and cystitis isolates, respectively.

**Supplementary Information:**

The online version contains supplementary material available at 10.1186/s12929-022-00848-5.

## Background

Urinary tract infections (UTI) are one of the commonest infections of humans, only second to respiratory tract infections in the rate of occurrence. The high morbidity of UTI, especially in reproductive age women, coupled with increased rates of antibiotic resistance among the commonly used UTI drugs, significantly contribute to increased healthcare costs for this condition [1], requiring effective strategies to manage it. In most UTI cases*, E. coli* is implicated, with 80–90% of uncomplicated UTIs caused by this organism in all age groups [2]. Consequently, most studies on UTI pathogenesis, and control strategies, have focussed on *E. coli*.

Uropathogenic *E. coli* (UPEC), the specialised *E. coli* strains that cause most UTIs, are endowed with specialised structures, molecules and regulatory systems, commonly referred to as virulence factors (VFs), that help the organism to colonize, invade and injure the host. Described UPEC VFs include diverse adhesins, toxins, siderophores, and surface polysaccharides and proteins, with over 40 suspected and confirmed VFs defined to date [3]. Adhesion of *E. coli* to host epithelial cells is an important initial and crucial step in UTI pathogenesis, and is effected through a wide variety of adhesins. One of the most described UPEC adhesins, and convincingly implicated in UTI pathogenesis, is P fimbria, which mediates Gal(α1-4)Gal-specific binding through the outermost part of the fimbria, the molecule PapG [4, 5]. The P fimbria consists of several subunits, PapA-G, which are encoded by a multicistronic gene cluster, the *pap* operon [6]. Many P fimbriated *E. coli* strains harbour 2 to three complete copies of the *pap* operon, with some having incomplete copies [6].

The *pap* gene cluster consists of 11 genes encoding; the main component of the fimbria rod (*papA*), *papEF*, which encodes adaptor subunits, and a terminal adhesin gene *papG*. The papG subunit occurs in 4 molecular variants, including classes I-IV, each encoded by a distinct allele of the adhesin gene *papG* [7, 8]. Each of these 4 papG molecular variants have distinct receptor binding specificities, and hence understood to confer differences in host range specificities [9–11] and /or capacity to cause specific UTI clinical syndromes [9, 12, 13], *albeit* some contradicting findings in other studies [14].

PapGII has been shown to be strongly associated with pyelonephritis in adult women and children [9, 15, 16], acute prostatitis in men [17], and bacteraemia in a wide range of hosts [18–20]. In contrast, the PapGIII variant has mostly been described in cystitis isolates in children, men and women [14, 21, 22]. PapGI is rarely encountered in the majority of UTI cases, being mostly detected in very small proportions in several host groups and clinical syndromes [14]. The PapGIV allele has been reported, but its distribution and function have not been well established [8]. However, there is still conflicting data from literature regarding the role played by the four PapG variants in host specificity, which probably arises from differences in study designs, population definitions, and geographical settings. Thus, whether the 4 PapG variants differ with respect to associated bacterial traits, clinical syndromes, or host characteristics, remains to be clearly defined in a stringently selected host population.

The *E. coli* population can be phylogenetically subdivided into 8 groups, namely A, B1, B2, C–F, and clade 1, as per the upgraded Clermont et al. molecular typing protocol [23]. Studies utilizing the initial molecular typing assay revealed that most *E. coli* extraintestinal pathogenic strains, including UPEC, derive from group B2 and to a lesser extent group D, and with phylogroups A and B1 mostly restricted to commensal and intestinal pathogenic *E. coli* strains [24–26]. There is limited data on the distribution of the newly described phylogenetic groups as per the revised molecular typing technique. Furthermore, few studies have analysed the relationship between *papG* genotypes and phylogenetic group status, to shed some light into the epidemiological relationships between the two.

Therefore, we studied urinary *E. coli* isolates from reproductive age women with UTI, and fecal isolates from healthy controls, from the same geographical area (Central West region, NSW, Australia) and time period, in order to define; (i) the *papG* class and phylogenetic group distribution among the isolates; (ii) the relationship between VF gene distribution and *papG* class type; (iii) the relationship between *papG* genotypes and phylogeny; and finally, (iv) the inferred virulence potential of cystitis and pyelonephritis isolates by *papG* allele genotype status.

## Materials and methods

### Study design and setting

This prospective study included 11 regional hospitals and 23 outpatient centers, in the Central West region of New South Wales (NSW), Australia. It was conducted with the help of volunteer physicians in the participating centers. A guiding protocol for urine collection and a strict clinical diagnostic criteria for defining cystitis or pyelonephritis, was given to each participating physician. Based on the medical history and patient physical examination, physicians recorded the following information in each patient recruited into the study; de-identified patient information: age, clinical UTI syndrome, previous UTI history, and any known underlying host conditions.

### *E. coli* strains and study subjects

Consecutively collected *E. coli* isolates (n = 601) from mid-stream urine (MSU) specimens of non-pregnant, reproductive-age (i.e., age 15–45 years) women with cystitis (n = 307) or, pyelonephritis (n = 294), as defined below, or from fecal specimens of healthy women without urinary symptoms (n = 235), were studied. Fecal controls were matched for sex and age within the overall age group. The isolates were collected over a 2-year period (June 2009-July 2011), with only one isolate per subject included in the study. To allow a focus on bacterial characteristics, with minimal confounding by host characteristics, patients with known diabetes mellitus, diarrhoea, antibiotic therapy in the last month, menstruation, or urinary tract abnormalities, were excluded. Urinary tract abnormality was defined based on the attending physician’s assessment at the time of the index patient encounter. Urological evaluation to exclude inapparent **s**tructural or functional genitourinary abnormalities was not done.

A diagnosis of cystitis or pyelonephritis required specific manifestations, as recorded by the treating medical practitioner based on a standardized protocol, and a MSU culture yielding ≥ 10^8^ cfu/L of *E. coli*. Cystitis-defining manifestations included dysuria, frequent urination, and/or suprapubic tenderness, without fever or loin pain. Pyelonephritis-defining manifestations included urinary symptoms plus, fever of ≥ 38 °C and flank pain, with or without nausea/vomiting.

Fecal control isolates were collected from consenting volunteers, who lacked UTI-associated manifestations as per self-report. These controls were matched with UTI subjects by age and, as closely as possible, by place of residence within the region. Each volunteer provided a written informed consent and a rectal swab. Rectal swabs were processed within 15 h of collection, and one arbitrarily chosen *E. coli* colony per specimen was used [27]. *E. coli* was identified by conventional biochemical tests, and the isolates were stored in 5% glycerol in trypticase soy broth at − 70 °C until further use.

### Ethics approval

The Sydney West Area Health Service (SWAHS) Human Ethics Research Committee and Charles Sturt University Ethics committee approved the study protocol. Guidelines for experimentation at the authors’ institutions were followed in the conduct of this clinical research. Since clinical information for patients with UTI was collected anonymously, patient consent was not obtained. However, written informed consent was obtained from each individual in the case of faecal specimens from healthy controls.

### Phylotyping and VF genotyping

*E. coli* phylogenetic groups were identified by the revised Clermont et al. method as previously described [23]. Twenty-two UPEC-associated VF genes (list in Table 1, and Additional file 1: Table S1), encompassing several VF functional categories, were detected using a previously described multiplex PCR-based reverse line blot (mPCR/RLB) hybridization assay [28]. The aggregate VF gene score was the sum of VF genes detected in an isolate, with multiple *pap* operon genes counted as one. Such VF gene scores have been shown to predict experimental in vivo virulence [29, 30].

**Table 1:** Distribution of *papG* alleles among 836 *Escherichia coli* isolates from reproductive-age women with pyelonephritis, cystitis, and healthy fecal controls

Trait^a^	Source of isolates, no.^b^ (%)			P value^c^		
	Pyelonephritis (n = 294)	Cystitis (n = 307)	Fecal (n = 235)	Fecal vs. Pyelo	Fecal vs. cystitis	Pyelo vs. cystitis
*papG* allele						
*papG1* only	0 (0)	4 (1)	0 (0)	NS^d^	NS	NS
*papGII* only	200 (68)	50 (16)	31 (13)	< 0.001	NS	< 0.001
*papGIII* only	12 (4)	93 (30)	45 (19)	< 0.001	0.002	< 0.001
*papGIV* only	0 (0)	4 (1)	0 (0)	NS	NS	NS
*papGI* + *papGII*	0 (0)	9 (3)	0 (0)	0.004	NS	0.004
*papGI* + *papGIII*	0 (0)	9 (3)	0 (0)	0.004	NS	0.004
*papGII* + *papGIII*	6 (2)	21 (7)	7 (3)	0.005	0.051	0.005
*papGI* + *papGII* + *papGIII*	3 (1)	0 (0)	0 (0)	NS	NS	NS
Any *papG* allele	221 (75)	190 (62)	83 (35)	< 0.001	< 0.001	< 0.001
No *papG* allele	73 (25)	117 (38)	152 (65)	< 0.001	< 0.001	< 0.001
*Phylogenetic group*						
A	8 (3)	2 (1)	52 (22)	< 0.001	< 0.001	0.058
B1	6 (2)	2 (1)	47 (20)	< 0.001	< 0.001	NS
B2	236 (80)	215 (70)	75 (32)	< 0.001	< 0.001	NS
C	3 (1)	16 (5)	2 (1)	NS	NS	NS
D	33 (11)	28 (9)	31 (13)	NS	NS	NS
E	3 (1)	16 (5)	7 (3)	NS	NS	NS
F	6 (2)	22 (7)	19 (8)	NS	NS	NS
Clade 1	0 (0)	6 (2)	2 (1)	NS	NS	NS

^a^Trait, either *papG* allele genotype or phylogenetic group

^b^No., number of isolates

^c^P values (by Fisher’s exact test) are shown where P < 0.05

^d^NS, not statistically significant, where P values are > 0.05

### Quality control

Each molecular testing was done in duplicate, and with independently prepared DNA lysates of the test isolates and with appropriate positive and negative controls.

### Statistical analysis

Comparisons of proportions was done by Chi-square or Fisher’s exact tests whilst VF scores were compared using the Mann–Whitney U-test. For comparisons of proportions for different characteristics in the same population, McNemar’s test was used. P values < 0.05 were considered significant.

## Results

### General distribution of *papG *alleles in *E. coli* isolates from reproductive-age women with cystitis or pyelonephritis and healthy fecal controls

The majority of pyelonephritis (75%) and cystitis (62%) isolates harboured at least one *papG* allele gene, as opposed to the lower proportion in fecal controls (35%) (P < 0.001 for each) (Table 1). The *papGII* gene was highly prevalent in pyelonephritis isolates (68%), whilst *papGIII* was the most abundant allele gene, *albeit* at a lower proportion, in cystitis isolates (30%), and the differences were highly statistically significant (P < 0.001). In contrast to both pyelonephritis and cystitis isolates, a significantly lower proportion of fecal isolates contained either only *papGII* (13%) or *papGIII* (19%) genes (P < 0.001), with most of the remainder devoid of any *papG* gene. The proportion of isolates harbouring a combination of *papG* allele genes was relatively small, ranging from 3% each in pyelonephritis and fecal isolates, to 13% in cystitis isolates. The gene *papGI* was relatively rare among the isolates, being detected as the only *papG* allele in 1% of the cystitis isolates, and in 3% each in combination with either *papGII* or *papGIII*, all in cystitis isolates. Likewise, the *papGIV* gene was rare, being detected in 1% of cystitis isolates only. The most common *papG* allele type combination was *papGII* and *papGIII*, being present in 2%, 7% and 3% of pyelonephritis, cystitis and fecal isolates, respectively (Table 1).

As previously observed, an overwhelming majority of pyelonephritis (80%) and cystitis (70%) isolates belonged to phylogenetic group B2, and a much lower proportion to group D at about 10% each for both clinical syndromes. In contrast to the UTI isolates, a much lower proportion of fecal isolates belonged to group B2 (32%), and a higher proportion to groups A (22%) and B1 (20%). However, the proportion of fecal isolates belonging to group D was almost equal to that in both pyelonephritis and cystitis isolates at 13%. The remaining phylogenetic groups were distributed variously different amongst the three groups, ranging from 1 to 8%.

### Distribution of *papG* alleles in relation to other VF genes among *E. coli* pyelonephritis isolates from reproductive-age women

On average, pyelonephritis isolates harboring *papG* genes also possessed a higher number of other VF genes, compared to their *papG* negative counterparts (Table 2). Specifically, all the VF genes tested, save for three (*afa*, *fyuA* and *kpsIII*), were detected in significantly higher proportions in *papG* positive isolates than negative ones. Consequently, VF gene scores were significantly higher in the *papG* positive isolates than in the negative ones (Table 2). In terms of specific *papG* alleles, *papGII* + isolates, which were the most prevalent in pyelonephritis isolates, on average harboured more VF genes than their *papGIII* counterparts, *albeit* small number of *papGIII* isolates analysed. Specifically, 12 of the 18 VF genes tested were much more prevalent in *papGII* + isolates than *papGIII* + ones. Genes that were much more prevalent in *papGIII* isolates than *papGII* included *afa* (25% vs 5%), *traT* (83% vs 70%), and *usp* (75% vs 66%). Consequently, VF gene scores were significantly higher in *papGII* than *papGIII* isolates. All the *papGII* positive pyelonephritis isolates harboured the *fimH* and *papC* genes, whilst no *gafD* and *bmaE* genes were detected in both *papGII* and *papGIII* isolates. Furthermore, no *kpsIII* gene was detected in *papGIII* isolates.

**Table 2:** Distribution of phylogenetic groups and virulence factor (VF) genes in relation to *papG* alleles among *E. coli* isolates from reproductive-age women with pyelonephritis

Trait	Number (%) of isolates				P value^d^	
Phylogenetic group or VF gene^a^	*papG* neg^b^ (n = 73)	*papG* pos^c^ (n = 221)	*papGII* + (n = 200)	*papGIII* + (n = 12)	*papG* neg vs. *papG* pos	*papGII* vs. *papGIII*
A	1 (1)	7 (3)	0 (0)	1 (8)	NS^e^	0.056
B1	2 (3)	4 (2)	0 (0)	1 (8)	NS	0.056
B2	47 (64)	189 (86)	196 (98)	7 (58)	0.005	< 0.001
C	1 (1)	2 (1)	1 (1)	1 (1)	NS	NS
D	18 (24)	15 (7)	4 (2)	3 (25)	< 0.001	0.004
E	1 (1)	2 (1)	1 (1)	1 (1)	NS	NS
F	2 (3)	4 (2)	2 (1)	2 (1)	NS	NS
Clade 1	0 (0)	0 (0)	0 (0)	0 (0)	NS	NS
*afa*	18 (25)	13 (6)	9 (5)	3 (25)	< 0.001	0.023
*bmaE*	0 (0)	4 (2)	0 (0)	0 (0)	NS	NS
*cnf1*	19 (26)	113 (51)	104 (52)	4 (33)	< 0.001	NS
*fimH*	67 (92)	221 (100)	200 (100)	10 (83)	< 0.001	0.003
*focG*	34 (46)	117 (53)	107 (54)	4 (33)	< 0.001	NS
*fyuA*	60 (82)	144 (65)	132 (66)	6 (50)	0.006	NS
*gafD*	0 (0)	3 (1)	0 (0)	0 (0)	NS	NS
*hlyA*	38 (52)	144 (65)	137 (69)	5 (42)	0.052	NS
*iutA*	48 (66)	190 (86)	182 (91)	8 (67)	< 0.001	0.025
*kpsII*	42 (58)	168 (76)	144 (72)	6 (50)	0.004	NS
*kpsIII*	7 (10)	18 (8)	18 (9)	0 (0)	NS	NS
*papAH*	42 (58)	199 (90)	189 (95)	10 (83)	< 0.001	NS
*papC*	45 (62)	212 (96)	200 (100)	9 (75)	< 0.001	< 0.001
*papEF*	44 (60)	212 (96)	196 (98)	10 (83)	< 0.001	0.039
*sfaS*	13 (18)	73 (33)	70 (35)	3 (25)	0.017	NS
*traT*	51 (70)	151 (76)	140 (70)	10 (83)	NS	NS
*ompT*	46 (63)	146 (73)	136 (68)	8 (67)	NS	NS
*usp*	50 (68)	143 (72)	132 (66)	9 (75)	NS	NS
^f,g^VF score (Median, range)	5 (1–9)	8 (3–15)	10 (3–15)	7 (2–10)	0.012	0.015

^a^The 22 virulence factors (VFs) analyzed were; *papA*, P fimbriae structural subunit; *papC*, P fimbriae assembly; *papEF*, fimbriae tip pilins; *papG,* P fimbriae adhesin (and alleles I, II and III); *sfaS*, S fimbriae; *focG*, F1C fimbriae; *afa/draBC*, Afimbrial adhesin (Dr-binding adhesin); *fimH*, type 1 fimbriae; *hlyA*, hemolysin; *cnf1,* cytotoxic necrotizing factor type1; *fyuA*, ferric yersiniabactin receptor; *iutA*, aerobactin receptor; *iroN*, catecholate siderophore receptor; *kpsMTII* group 2 capsule (with K1 and K2 variants); *kpsMTIII*, group 3 capsule; *traT*, serum-resistance associated; *ompT*, outer membrane protein T (protease); *bmaE*, M fimbriae; *gafD*, (G) fimbriae

^b^*papG* neg, *papG* gene negative (for all alleles)

^c^*papG* pos, *papG* gene positive (for any allele)

^d^P values (by Fisher’s exact test) are shown where P < 0.05

^e^NS, not statistically significant, P > 0.05

^f^VF score, sum of all VF genes tested, with *pap* operon genes counted as a unit

^g^P values by Mann–Whitney test are shown where P < 0.05

In terms of the relationship between *papG* alleles and phylogeny, a greater proportion of *papG* positive isolates (86%) was restricted to group B2 compared to 64% for *papG* negative isolates (P = 0.006). Almost all *papGII* + isolates (98%) were of phylogenetic group B2 compared to only 58% for *papGIII* + isolates (P < 0.001). Based on the group D status, a greater proportion (25%) of *papGIII* + isolates belonged to this group compared to only 2% for *papGII* isolates, *albeit* very small number of *papGIII* + isolates (n = 12) (P = 0.004). None of the *papGII* + isolates belonged to phylogenetic groups A, B1, Clade 1, and only 1 *papGIII* isolate each, belonged to these 3 phylogenetic groups. The recently described phylogenetic groups (E, F and clade 1) were evenly distributed amongst *papG* negative vs. *papG* positive isolates, and *papGII* + vs. *papGIII* + isolates, but with very small numbers of isolates in each subgroup.

### Distribution of *papG* alleles among *E. coli* cystitis isolates from reproductive-age women

Similar to pyelonephritis isolates, on average, cystitis *papG* positive isolates harboured more VF genes than their *papG* negative counterparts, with only one gene (*afa*) being statistically much more abundant in the *papG* negative isolates at 20% vs. 5% (P < 0.001). Likewise*, papGII* + isolates harboured more VF genes compared to *papGIII* + ones, with only 2 genes (*afa*, *usp*) being much more prevalent in the *papGIII* isolates, *albeit* statistically insignificant (P > 0.05), and hence VF gene scores were significantly lower in *papGIII* isolates compared to *papGII* ones (Table 3). Due to the high number of isolates harbouring both the *papGII* and *papGIII* genes concurrently among cystitis isolates (n = 21), we analysed them further for VF gene carriage distribution. These isolates harboured more VF genes than any other isolates, with an overwhelming majority of the isolates harbouring most of the tested genes. Specifically, at least 77% of these isolates containing both *papGII* and *papGIII* genes harboured 14 of the 18 VF genes analysed. As a result, the VF gene scores of these isolates were the highest among all the isolates studied, pyelonephritis and fecal isolates included. Furthermore, these isolates with a combination *papGII* and *III* allele genotype carried the following genes*, fimH*, *focG, papAH*, *papC*, *papEF* and *traT.* The gene *bmaE*, which was not detected in any pyelonephritis isolates, was detected in 5% of *papGIII* isolates only and none in *papGII* and *papGII* + *papGIII* positive isolates.

**Table 3:** Distribution of phylogenetic groups and virulence factor (VF) genes in relation to *papG* alleles among *E. coli* isolates from reproductive-age women with cystitis

Trait	Number (%) of isolates						P value^d^	
Phylogenetic Group or VF^a^ gene	*papG* neg^b^ (n = 117)	*papG* pos^c^ (n = 190)	*papGII* + (n = 52)	*papGIII* + (n = 95)	*papGIV* + (n = 4)	*papGII* + *papGIII* (n = 21)	*papG* neg vs. *papG* pos	*papGII* vs *papGIII*
A	2 (2)	0 (0)	0 (0)	0 (0)	0 (0)	0 (0)	^e^NS	NS
B1	2 (2)	0 (0)	0 (0)	2 (2)	0 (0)	0 (0)	NS	NS
B2	82 (70)	149 (78)	52 (100)	86 (91)	0 (0)	21 (100)	0.037	0.027
C	6 (5)	4 (2)	2 (4)	4 (4)	2 (5)	0 (0)	NS	NS
D	13 (11)	14 (7)	2 (4)	12 (13)	0 (0)	1 (5)	NS	NS
E	7 (6)	7 (4)	1 (2)	4 (4)	1 (25)	0 (0)	NS	NS
F	3 (3)	12 (6)	1 (2)	5 (5)	1 (25)	1 (5)	NS	NS
Clade 1	2 (2)	4 (2)	1 (2)	1 (1)	0 (0)	0 (0)	NS	NS
*afa*	23 (20)	10 (5)	0 (0)	3 (3)	0 (0)	9 (42)	< 0.001	NS
*bmaE*	0 (0)	0 (0)	0 (0)	5 (5)	0 (0)	0 (0)	NS	NS
*cnf1*	22 (19)	108 (57)	23 (44)	35 (37)	2 (50)	18 (86)	< 0.001	NS
*fimH*	108 (92)	190 (100)	52 (100)	93 (98)	4 (100)	21 (100)	< 0.001	NS
*focG*	42 (36)	78 (41)	18 (35)	21 (22)	1 (25)	21 (100)	NS	NS
*fyuA*	73 (62)	110 (58)	26 (50)	39 (41)	2 (50)	18 (86)	NS	NS
*gafD*	0 (0)	0 (0)	0 (0)	0 (0)	0 (0)	2 (9)	NS	NS
*hlyA*	45(38)	105 (55)	35 (67)	44 (46)	2 (50)	18 (86)	0.005	0.016
*iutA*	63 (54)	139 (73)	44 (85)	49 (52)	2 (50)	19 (92)	< 0.001	< 0.001
*kpsII*	47 (40)	116 (61)	30 (58)	30 (32)	2 (50)	16 (77)	0.003	0.003
*kpsIII*	6 (5)	6 (3)	2 (4)	0 (0)	0 (0)	(23)	NS	NS
*papAH*	78 (67)	156 (82)	48 (92)	77 (81)	3 (75)	21 (100)	0.002	NS
*papC*	77 (66)	162 (85)	49 (94)	74 (78)	3 (75)	21 (100)	< 0.001	NS
*papEF*	75 (64)	154 (81)	49 (94)	77 (81)	3 (75)	21 (100)	0.001	NS
*sfaS*	21 (18)	63 (33)	18 (35)	25 (26)	1 (25)	16 (77)	0.004	NS
*traT*	85 (73)	148 (78)	37 (72)	77 (78)	3 (75)	21 (100)	NS	NS
*ompT*	78 (67)	137 (72)	32 (62)	60 (63)	3 (75)	17 (82)	NS	NS
*usp*	81 (69)	135 (71)	30 (58)	66 (69)	3 (75)	18 (86)	NS	NS
^f,g^VF score (Median, range)	3 (1–7)	5 (2–13)	7 (3–12)	5 (1–10)	6 (1–9)	14(10–13)	0.001	0.016

^a^The 22 virulence factors analyzed were; *papA*, P fimbriae structural subunit; *papC*, P fimbriae assembly; *papEF*, fimbriae tip pilins; *papG,* P fimbriae adhesin (and alleles I, II and III); *sfaS*, S fimbriae; *focG*, F1C fimbriae; *afa/draBC*, Afimbrial adhesin (Dr-binding adhesin); *fimH*, type 1 fimbriae; *hlyA*, hemolysin; *cnf1,* cytotoxic necrotizing factor type1; *fyuA*, ferric yersiniabactin receptor; *iutA*, aerobactin receptor; *iroN*, catecholate siderophore receptor; *kpsMTII* group 2 capsule (with K1 and K2 variants); *kpsMTIII*, group 3 capsule; *traT*, serum-resistance associated; *ompT*, outer membrane protein T (protease); *bmaE*, M fimbriae; *gafD*, (G) fimbriae

^b^*papG* neg, *papG* gene negative (for all alleles)

^c^*papG* pos, *papG* gene positive (for any allele)

^d^P values (by Fisher’s exact test) are shown where P < 0.05

^e^NS, not statistically significant, P > 0.05

^f^VF score, sum of all VF genes tested, with *pap* operon genes counted as a unit

^g^P values by Mann–Whitney test are shown where P < 0.05

Similar to pyelonephritis isolates, the majority (75%) of cystitis isolates belonged to phylogenetic group B2, with a greater proportion of *papG* positive isolates (78%) belonging to this phylogenetic group, as opposed to 70% for *papG* negative ones. Specifically, all the *papGII* isolates, and 91% of *papGIII* isolates belonged to this group. For group D status, a greater proportion of *papGIII* isolates (13%) belonged to this group compared to only 4% for *papGII* isolates, *albeit* statistically insignificant. The rest of the phylogenetic groups were generally evenly distributed amongst the different subgroups.

### Distribution of *papG *genotypes among *E. coli* pyelonephritis and cystitis isolates from reproductive-age women

An overwhelming majority of pyelonephritis isolates harbouring only the *papGII* gene (79%), were positive for most of the other *pap* genes tested, compared to only 17% for isolates carrying the *papGIII* gene only (P < 0.001) (Table 4). The remainder of the isolates that harboured only the *papGII* gene also harboured at least 2 of the 3 other *pap* operon genes tested. All the pyelonephritis isolates containing only the *papGII* gene, had at least one other *pap* operon gene detected. For isolates harbouring only the *papGIII* gene, the prevalence of other *pap* genes was generally similar, mostly at 17% each. All the isolates with a combination genotype of *papGII* and *papGIII* genes possessed all the other *pap* genes tested. Most (82%) of the *papG* negative pyelonephritis isolates also lacked the other *pap* genes tested, with the remainder harbouring only one of the other *pap* operon genes.

**Table 4:** Distribution of *pap* genotypes among uropathogenic *Escherichia coli* isolates from pyelonephritis

Trait	Number (%) of isolates				^b^P value
Pap genotype	*papGII* + only (n = 200)	*papGIII* + only (n = 12)	*papGII* + *papGIII* (n = 6)	*papG* neg^a^ (n = 73)	*papGII vs. papGIII*
*papEF*^+^*papAH*^+^*papC*^+^	158 (79)	2 (17)	6 (100)	0 (0)	< 0.001
*papEF*^+^*papAH*^+^*papC*^−^	22 (11)	2 (17)	0 (0)	0 (0)	NS
*papEF*^−^*papAH*^−^*papC*^+^	0 (0)	2 (17)	0 (0)	6 (8)	0.003
*papEF*^−^*papAH*^−^*papC*^−^	0 (0)	2 (17)	0 (0)	60 (82)	0.003
*papEF*^−^*papAH*^+^*papC* +	16 (8)	2 (17)	0 (0)	0 (0)	NS
*papEF*^+^*papAH*^−^*papC*^−^	0 (0)	1 (8)	0 (0)	7 (10)	NS
*papEF*^−^*papAH*^+^*papC*^−^	4 (2)	1 (8)	0 (0)	0 (0)	NS

^a^*papG* neg, *papG* gene negative (for all alleles)

^b^P values (by Fisher’s exact test) are shown where P < 0.05

For cystitis isolates, *papGI* isolates, which were solely confined to this group, belonged only to one *pap* genotype, *papEF*^*−*^*papAH*^+^*papC*^*−*^, *albeit* only 4 isolates (Table 5). The majority of cystitis isolates possessing the *papGII* gene only (46%), contained all the *pap* operon genes tested, and hence assumed to have an intact or complete *pap* operon. The majority of the remaining isolates in this group possessed at least 2 of the other 3 *pap* genes tested. Cystitis isolates harbouring only the *papGIII* allele gene, mostly (38%) also contained all the operon genes tested, or harboured at least 2 other *pap* genes (32%). For cystitis isolates harbouring both *papGII* and *papGIII* genes concurrently, most (82%) also contained all the *pap* genes tested. Likewise, most (65%) of the cystitis isolates devoid of any *papG* allele genes were also null for other *pap* operon genes.

**Table 5:** Distribution of *pap* genotypes among uropathogenic *Escherichia coli* isolates from cystitis

Trait	Number (%) of isolates					^b^P value
Pap operon genotype	*papGI* only (n = 4)	*papGII* only (n = 52)	*papGIII only* (n = 86)	*papGII* + *papGIII* (n = 22)	*papG* neg^a^ (n = 132)	*papGII vs papGIII*
*papEF*^+^*papAH*^+^*papC*^+^	0 (0)	24 (46)	33 (38)	16 (82)	18 (14)	NS
*papEF*^+^*papAH*^+^*papC*^−^	0 (0)	11 (21)	15 (17)	2 (9)	9 (7)	NS
*papEF*^−^*papAH*^−^*papC*^+^	0 (0)	0 (0)	10 (12)	0 (0)	2 (2)	0.013
*papEF*^−^*papAH*^−^*papC*^−^	0 (0)	0 (0)	0 (0)	0 (0)	86 (65)	NS
*papEF*^−^*papAH*^+^*papC* +	0 (0)	10 (19)	13 (15)	2 (9)	7 (5)	NS
*papEF*^+^*papAH*^−^*papC*^−^	0 (0)	3 (6)	3 (4)	0 (0)	6 (5)	NS
*papEF*^−^*papAH*^+^*papC*^−^	4 (100)	4 (8)	12 (14)	2 (9)	4 (3)	NS

^a^*papG* neg, *papG* gene negative (for all alleles)

^b^P values (by Fisher’s exact test) are shown where P < 0.05

## Discussion

Our aim was to understand the role played by *papG* alleles in UTI pathogenesis, specifically their association with particular UTI syndromes, other VF genes and phylogenetic groups. Our findings demonstrate, in a stringently selected reproductive age women population by region, time and UTI syndrome, that *papGII* allele is strongly associated with pyelonephritis, with 68% of the isolates harboring this gene, whilst *papGIII* was more common in cystitis isolates, *albeit* at a lower level of influence, with a majority of 31% of the cystitis isolates harboring this allele only. These findings are in agreement with those of several other studies in different jurisdictions [9, 10, 31]. However, despite the stringent selection of the isolates for inclusion in the present study according to the uro-clinical category, it is possible that some misclassification of the uro-clinical syndrome might have occurred.

The predominance of the *papGII* gene in the studied pyelonephritis isolates, is in line with the demonstrated abundance of papGII iso-receptors in human kidney tissue, indicating an important role for this allele in ascending UTI infection as previously observed [32]. The *papGII* gene has been associated epidemiologically with pyelonephritis and urinary-source bacteremia in directed, usually PCR-based studies [33], and was demonstrated experimentally, with varying degrees of rigor, to contribute to kidney infection in murine and monkey models [34–36]. However, contrary to these findings, is the reported dominance of *papG* class II gene in pediatric cystitis isolates, *albeit* small number of isolates studied [37, 38], suggesting that associations of *papG* alleles with specific clinical syndromes may depend on the specific population studied, including age, gender, and geographical locale [37, 38]. It is thus important that comparisons of studies take into consideration VF gene carriage population dynamics.

On average, pyelonephritis and cystitis isolates, irrespective of *papG* allele gene status, more often belonged to phylogenetic group B2, and to a lesser extent group D, had higher VF gene scores, than fecal isolates which were mostly confined to groups A and B1. Our results are consistent with previous evidence of the domination of *E. coli* phylogenetic group B2 among extraintestinal pathogenic *E. coli* (EXPEC), and suggest that phylogenetic group B2 may be the main source, and hence presumably the original source of many VF genes in EXPEC [39]. These EXPEC VF genes are understood to be mainly inherited vertically within evolutionary ancestries, but can also be transferred horizontally between lineages, on gene bocks that contain multiple contiguous VF genes, commonly referred to as “pathogenicity-associated islands” (PAIs), or through plasmids [9, 39]. Although on average the UTI isolates had higher VF gene scores than the fecal isolates as previously observed [28, 40–42], the pyelonephritis isolates contained more individual VF genes, and consequently had higher aggregate VF gene scores, than the cystitis (or fecal) isolates, suggesting that VF gene repertoire plays a significant role in ascending UTI pathogenesis [43].

VF gene distribution by *papG* gene status revealed that *papG* positive pyelonephritis isolates on average contained more VF genes, and hence higher VF scores, than their *papG* negative counterparts, implying an association of *papG* with several other VF genes. This may be due to the fact that the *pap* operon can be located on chromosomal or PAIs which contain other VF genes, and hence are transmitted as a block of VF genes [6, 44]. The high VF gene carriage amongst the pyelonephritis isolates was mostly due to the contribution of *papGII* + isolates, which on average carried more VF genes than their *papGIII* counterparts as evidenced by higher VF scores, with 16 out of 18 VF genes analysed being much more abundant in the *papGII* isolates. This suggests an association of *papGII* with a wide variety of VF genes, compared to *papGIII*, and hence a possible increased inferred virulence potential of such strains, and increased capacity to cause ascending UTI. Likewise, the overall trend for cystitis isolates in relation to *papG* gene *vs*. other VF gene carriages, followed a similar pattern to pyelonephritis isolates. Taken together, these results strongly suggest that *papG* is an important VF gene in UTI pathogenesis, especially given that it is involved in the attachment of *E. coli* to the epithelial cells of the urogenital tract [45].

Interestingly, although *papGIII* gene was the most prevalent allele in cystitis isolates (31%), on average, *papGII* cystitis isolates had significantly higher VF gene scores (P = 0.03), suggesting increased inferred virulence potential for such strains as also demonstrated in the pyelonephritis cohort. This suggests an increased association of *papGII* with a wide range of VF genes, possibly due to their phylogenetic group B2 status as most of the *papGII* isolates derived from this phylogenetic group. However, even among the *papGII* isolates, pyelonephritis isolates had higher overall VF gene carriage than cystitis ones (data not shown), probably indicating presence of *papGII* clones within phylogenetic group B2, with differing levels of virulence, based on other factors, including possibly VF gene copy number, and other host and bacterial factors, as previously observed by us [41, 42]). Notably, predominance of the *papGII* allele among avian pathogenic *E. coli* isolates with high homology to human isolates has been previously reported [46], and also similarity between human and avian *E. coli* strains representing zoonotic potential has been demonstrated [47], suggesting that horizontal gene transfer of pathogenicity elements from chickens to humans may play a role in UTI pathogenesis [48].

Since we had a reasonable number of cystitis isolates that had a combination genotype of *papGII* and *papGIII* genes concurrently (n = 21), we analysed these isolates in relation to VF gene carriage. These isolates on average had the highest VF gene scores, with all VF genes tested, save for two (*gafD* and *sfaS*), having a prevalence of at least 67% each in these isolates. Hence, as per murine and mice studies [29], the inferred virulence capacity for such isolates was very high, and as such, we expected these strains to be confined to the pyelonephritis group, which was not the case. A further analysis of the patients from which the isolates originated revealed that they were all early UTI cases, suggesting that the bacterial strain might not have had enough time to cause ascending UTI. However, it is rather surprising that we did not find a high number of isolates containing both alleles amongst the pyelonephritis isolates.

We further analysed the association between the different *papG* gene alleles and other *pap* operon genes since they are encoded on the same *pap* operon. Among pyelonephritis isolates, *papG* negative isolates were more likely to be negative for all other *pap* genes tested (82%), suggesting complete absence of the *pap* operon or possible deletion [49], and hence inability to produce the P fimbria, or only positive for *papC* (8%), suggesting presence of an incomplete or truncated *pap* operon and hence incapacity to express P fimbria. This finding indicates that other VF adhesin genes or other bacterial or host factors may also be involved in ascending UTI pathogenesis, *albeit* on a smaller scale.

In the case of cystitis isolates, the majority (65%) of *papG* negative isolates, *albeit* lower proportion compared to the pyelonephritis isolates (82%), harbored no other *pap* genes, and hence were presumed to lack capacity to express P fimbriae. This finding suggests that for the pathogenesis of cystitis, other adhesins may be involved, including *afa*, *sfaS*, *fimH*, *bmaE*, *gafD*. Indeed, when these isolates were analysed for other adhesins, they, on average harbored a higher and wider variety of adhesins compared to their *pap* operon positive counterparts (data not shown). Many microorganisms have the genetic capacity to express different adhesins, providing access to multiple receptors and therefore increasing their pathogenicities [50]. Specifically, about 14% of our *papG* negative cystitis isolates were positive for all *pap* genes tested, suggesting possible deletion of the *papG* gene or presence of a yet to be described *papG* allele.

The majority of *papGII* positive pyelonephritis isolates (79%) were also positive for all the other *pap* operon genes tested, and hence had the ability to express P fimbriae under the right conditions [51]. In contrast, the pyelonephritis *papGIII* gene isolates demonstrated a limited capacity to express P fimbriae, as only 17% of the isolates presumably carried a complete *pap* operon, whilst the rest had incomplete operons with basically a uniform spread of other *pap* operon genes, mostly at 17% for each of the individual *pap* genes tested. This probably explains why *papGIII* isolates were more likely to be detected in cystitis isolates, as such isolates would have lacked the capacity to produce P fimbriae which is considered important in ascending UTI pathogenesis. It is not clear why the *papGII* gene was associated with a complete *pap* operon in pyelonephritis isolates, which calls for further studies to investigate this finding.

When compared by *papG* allele status, the majority of cystitis *papGII* + (46%) and *papGIII* + (38%) isolates contained all the other *pap* genes tested, and hence considered to have the capacity to express P fimbria. However, this was much lower than was the case with pyelonephritis isolates, where an overwhelming majority of the *papGII* strains had a presumed complete *pap* operon. These findings again seem to highlight that the capacity to express P fimbria is important in ascending UTI pathogenesis as observed by others [52, 53]. Furthermore, a large majority of isolates that contained both alleles concurrently (82%), contained a presumed intact *pap* operon. Although such isolates were, in the present study, confined to the cystitis isolates, we think that they had the capacity to cause ascending UTI over time as most of them were limited to early UTI cases.

Although the above argument that *papGII* operons from pyelonephritis isolates are more likely to be complete than *papGII* or *papGIII* operons from cystitis isolates, is plausible from a pathogenicity perspective, caution must be exercised in the interpretation of the findings. Firstly, the interpretations are based on the amplification of a limited region covering about 1 kb of the 9 kb *pap* operon, which limits the strength of the argument about the completeness of the *pap* operon. It is reasonable to argue that insertions or other recombination events are more likely to occur in the rest of the 8 kb region that was not investigated. Furthermore, some minor sequence variants have been described in the primer binding regions of *papA* and *papE* genes, and thus one can also hypothesize that such variants could have been present in the negative isolates. However, from an epidemiological viewpoint, we think that such minor variants would not have much effect on the overall picture.

Although our findings seem to suggest association of specific *papG* alleles with specific UTI syndromes, contrasting findings, such as the presence of strains with a combination of both *papGII* and *papGIII* genes amongst cystitis strains, and presence of a significant proportion of *papGII* isolates among cystitis strains, suggest that epidemiologic associations between individual *papG* alleles and specific clinical syndromes must be interpreted cautiously because such associations may be due primarily to other bacterial properties that are differentially linked with particular *papG* allele, possibly as part of a PAI rather than to any specific pathogenetic role of the *papG* allele itself. Like many other *E. coli* VF genes, the *pap* operon encoding P fimbria lies on PAIs [9, 54], which are large horizontally transferable genetic elements assumed to play an important role in the evolution of pathogenic *E. coli* [9, 54].

In 25% and 38% of pyelonephritis and cystitis isolates, respectively, both the major subunit gene (*papA*) and the distal adhesin gene (*papG*) were absent, suggesting presence of truncated P fimbrial operons as previously observed [55] or presence of minor variants. At least 95% of the *papGII* pyelonephritis isolates were positive for both *fimH* and other *pap* operon genes tested, and about 50% of them were positive for *pap* operon genes, *fimH*, and *sfa*/*foc*, implying capacity of these strains to express a wide variety of adhesins. This is in agreement with studies demonstrating that type 1, P, S, F1C, and Dr fimbriae, attach to different sites within the human kidney [32], and thus strains endowed with a diverse range of fimbrial types are more likely to have overall success during renal colonization [32]. Furthermore, P and type 1 fimbriae appear to act in synergy to promote colonization of kidney [56].

Although the *papGI* allele is relatively rare amongst clinical urinary isolates, it was surprisingly detected in 7% of the present isolates as follows; as the only *papG* allele in 1% of the cystitis isolates; in 3% each in combination with either *papGII* or *papGIII*, and finally as part of a concurrent combination with both *papGII* and *papGIII* genes in 1% of pyelonephritis isolates. To the best of our knowledge, this is one of the few, if any, studies in which the *papGI* allele was this much abundant, and may be attributed to differences in the distribution of *papG* alleles by geographical location, population cohort or other yet to be elucidated factors. The genetic make-up, or other aspects of the *papGI* + strains need to be more specifically determined to see if they belong to the same clonal group. Similarly, the *papGIV* gene, which has been rarely described in previous studies, was detected in only 1% of our isolates, and was only confined to the cystitis subgroup. More studies are needed to clarify the role of this *papG* allele in UTI pathogenesis.

The identification of a small proportion of cystitis isolates (3%) with the *papGI* and *papGIII* allele class combination highlights that this *papG* genotype, although rarely encountered among clinical isolates, is not limited to source strain J96 as was previously assumed [57, 58]. Previous reports indicate that this genotype is characteristic of a J96-like clonal group of *E. coli* strains of serotype O4:H5:F13 [55, 58], and some of these strains have caused cystitis, pyelonephritis, urosepsis, and bacteraemia of unknown origin, in parts of Europe and the United States of America (USA) [37, 38]. Both the *papGI* and *papGIII* alleles are associated with a *papA* molecule of the F13 serotype [55, 58], and may explain the present finding in which isolates that were *papGI* allele positive, also contained the *papAH* gene.

Strengths of this study include the large number of well-characterized cystitis, pyelonephritis, and fecal isolates, from the same geographical region and time period. This is important since human-associated *E. coli* strains can vary dramatically by region and over time [59, 60]. Other strengths include the extensive array of bacterial traits studied and the analysis of their distribution by phylogenetic group and uro-clinical syndrome.

Study limitations include the use of multiple comparisons, which can increase the chance of type 1 errors [61]. However, we regard our study as being of an exploratory nature rather than definitive, requiring future confirmatory studies, and is also designed to generate further hypotheses for future studies. Furthermore, virulence level was inferred based on molecular attributes, not in vivo assessment, and presence-absence testing for a defined set of VF genes can overlook other potentially important determinants of cystitis and pyelonephritis, including unrecognized VF genes [62], minor sequence variants of described VF genes [63, 64], or differences in VF gene expression [65]. Another limitation of the study is the possible overlap in the classification of isolates by uro-clinical syndrome into the 2 groups of cystitis and pyelonephritis. However, this is somehow compensated for by the large number of isolates studied. And finally, due to limited budget, whole genome sequencing was not performed, which could have shed some light into possible association of *papG* alleles with specific clones, presence of novel alleles, and whether the *pap* operons were truly disrupted.

## Conclusions

Findings from this study suggest that the association of the *papGII* gene with several other VF genes may explain its predominance in *E. coli* strains from pyelonephritis cases, as opposed to the predominance of the *papGIII* gene in cystitis cases.

## Supplementary Information

**Additional file 1: Table S1.** Novel oligonucleotide probes and primers developed for the mPCR/RLB assay for detection of uropathogenic *E. coli* virulence factor genes.

## Data Availability

The datasets and range of bacterial isolates analysed are available from the corresponding upon reasonable request.
