# Supplementary material for: Distribution of papG alleles among uropathogenic Escherichia coli from reproductive age women
Source: J Biomed Sci. 2022 Sep 7;29:66. doi: 10.1186/s12929-022-00848-5 (PMC9450305; doi:10.1186/s12929-022-00848-5)
Supplement: Supplementary file 1 — Additional file 1: Table S1. Novel oligonucleotide probes and primers developed for the mPCR/RLB assay for detection of uropathogenic E. coli virulence factor genes. [file 12929_2022_848_MOESM1_ESM.docx]

**Table S1. Novel oligonucleotide probes and primers developed for the mPCR/RLB assay for detection of uropathogenic *E. coli* virulence factor genes**

| **Probe/Primer name^a^** | **Length (bp)** | **Tm (^o^C)** | **GenBank**  **Accession no.** | **Sequence (5’to 3’) and coordinates within GenBank entry** |
| --- | --- | --- | --- | --- |
| papC-AP | 20 | 63 | X61239 | 4817 GTAGCCGGCCATATTCACAT 4798 |
| papC-SP | 20 | 64 | X61239 | 4924 TAGTCCGCTGGCAAATTTGT 4944 |
| fimH-AP | 25 | 61 | AJ225176 | 1864 CACATCATTATTGGCGTAAATATTC 1840 |
| fimH-SP | 25 | 60 | AJ225176 | 2231 GTTTATCAATAAAGAAATCA CAGGG 2255 |
| cnf1-AP | 28 | 61 | X70670 | 1700 TTTTCTATTGCATGTAGAACAGAATTTA 1673 |
| cnf-1-SP | 26 | 65 | X70670 | 2097 CCAG GAGGTACTTAGCAGCGTTATAA 2122 |
| hlyA-AP | 21 | 62 | M10133 | 2570 AAACATTGCCTGTTTTGAAGC 2550 |
| hlyA-SP | 23 | 60 | M10133 | 3549 GGTGATGACCATATAGAAGGAAA 3571 |
| papG I-III-AP | 23 | 65 | M20181 | 1244 AAAGCTGGGAACCATTTTTTCAT 1222 |
| papG I-III-SP | 21 | 65 | M20181 | 2209 GCAACGCTGCTCATGATATTG 2229 |
| papG I-AP | 22 | 64 | X61239 | 8913 CCTGAAAATACACTCCACTCGC 8892 |
| papG I-SP | 26 | 60 | X61239 | 9285 GCCACTAATACATTGATGTTATCATT 9310 |
| papG II-AP | 22 | 60 | M20181 | 1645 GTTTCTGACACAAATTACCTGC 1624 |
| papG II-SP | 20 | 65 | M20181 | 1755 CGGCATACAGCGTCATTTCG 1774 |
| papGIII-AP | 23 | 61 | X61238 | 1464 TCAGACCAGTAAAGACCATGAGT 1442 |
| papGIII-SP | 24 | 61 | X61238 | 1632 GTTTCAATATCGGAGAAAAAAGAA 1655 |
| PapGIV-AP | 23 | 60 | AF304159 | 97 GACTATTCTGGTTATGATTC 116 |
| PapGIV-SP | 22 | 60 | AF304159 | 453 CAATGAATTAAGGTTTAG 470 |
| afa/draBC-AP | 23 | 65 | X76688 | 4632 CGGCGTCGGGTTAACCCCCTTCA 4610 |
| afa/draBC-SP | 21 | 65 | X76688 | 5139 CTGAAGACCTGTCTGACCCGT 5159 |
| focG-AP | 23 | 62 | S68237 | 678 TACCCTCCCTGTAACAGTAATCG 656 |
| focG-AP | 26 | 62 | S68237 | 947 TTGGTTCAACAAAA GTTGTTACAGTG 972 |
| sfaS-AP | 23 | 64 | S53210 | 718 GTTCTTTGCAAAACATTACCCGT 697 |
| sfaS-SP | 21 | 63 | S53210 | 875 GGATGGGCAGACATACTATGC 895 |
| fyuA-AP | 20 | 62 | Z38064 | 814 GTGCCGCCTAAGTCATCGCT 795 |
| fyuA-SP | 20 | 62 | Z38064 | 1519 AGGGATATAAACCTTCCGGG 1538 |
| iutA-AP | 20 | 60 | X05874 | 892 ATTCATCGATGTTCAGCGTA 873 |
| iutA-SP | 21 | 64 | X05874 | 1111 TACTACCGCGATGAGTCGTTG 1131 |
| traT-AP | 23 | 63 | J01769 | 505 CTCAAGGTTACGCTTCTTGATTG 482 |
| traT-SP | 22 | 65 | J01769 | 706 GATAAGATGGATCTGCGGGAGT 727 |
| papAH-AP | 25 | 62 | X61239 | 1842 GGAATAGTTGGAGCAGCATTATTTA 1818 |
| papAH-SP | 20 | 64 | X61239 | 2475 CCCTTCCTGAATACTGGGGA 2494 |
| papEF-AP | 24 | 63 | X61239 | 8104 CATTTTATTTTACGAGATATAAAATTAACG 8075 |
| papEF-SP | 24 | 62 | X61239 | 8317 AAAAACCATAAGCATATCCTGTCC 8340 |
| bmaE-AP | 23 | 62 | M15677 | 123 TGTATGAGTGGCTGTTACTGTCA 101 |
| bmaE-SP | 23 | 62 | M15677 | 539 GACGGAAAATTAACCTGATGAAA 561 |
| gafD-AP | 21 | 61 | L33969 | 153 ATTGCATGAGTGCTGGAATAA 132 |
| gafD-AP | 27 | 60 | L33969 | 1013 GAATATCAACATTTACGTTTTCCTATC 1039 |
| iroN-AP | 20 | 63 | AF135597 | 1770 GGTTTGGGGCTTTAAAGGTT 1751 |
| iroN-SP | 20 | 61 | AF135597 | 2341 TGGGGACGAACTTCAATTAC 2360 |
| ompT-AP | 20 | 64 | X06903 | 648 GTCGAGTTGACTGACTTTTCG 628 |
| ompT-SP | 21 | 61 | X06903 | 1126 TGGTGGAATCATCTGATAAC 1146 |
| usp-AP | 20 | 65 | AB056434 | 6863 CTTCACCCGTATGAACACCAT 6843 |
| usp-SP | 20 | 63 | AB056434 | 7201 GCTGCCTGGTGTTGTAACAG 7220 |
| kpsII-AP | 23 | 64 | X53819 | 340 TGCATAATGTAACCAAAAATGCC 318 |
| kpsII-SP | 29 | 64 | X53819 | 516 ACGCTGATTTACGTTGCTGTTTATATATT 544 |
| kpsIII-AP | 24 | 62 | AF007777 | 4095 CCATAAAAACCAATGCTGATATTG 4072 |
| kpsIII-SP | 25 | 60 | AF007777 | 4393 CAGGAAAA TCTACTTTGTTACGTTT 4417 |
| *E.coli* 16S-AP | 20 | 20 | J01859 | 1210 AGGGCCATGATGACTTGACG 1191 |
| *E.coli* 16S-SP | 23 | 23 | J01859 | 1458 GCGCTTACCACTTTGTGATTCAT 1480 |
| *E.coli* 16S-AB | 19 | 60 | J01859 | 1539 AGGAGGTGATCCAACCGCA 1521 |
| *E.coli* 16S-SB | 20 | 62 | J01859 | 1169 AACTGGAGGAAGGTGGGGAT 1188 |

^a^The suffix B indicates a biotin-labeled primer, and P indicates an amine-labeled probe. “A”, antisense; “S”, sense.
